# Coaching effectiveness in competitive youth contact sports and martial arts: athletes’ and coaches’ perceptions

**DOI:** 10.3389/fpsyg.2025.1725858

**Published:** 2026-01-09

**Authors:** İlayda Müjdeci, Koray Kılıç, Aykut Bulut, Hakan Kuru

**Affiliations:** 1 Faculty of Sport Sciences, Ahi Evran University, Kırşehir, Türkiye; 2 Faculty of Education, Ahi Evran University, Kırşehir, Türkiye; 3 Faculty of Sports Sciences, Istanbul Rumeli University, Istanbul, Türkiye

**Keywords:** athlete development, program evaluation, relative age effects, the 4 Cs, youth sport

## Abstract

**Background:**

Contact sports and martial arts can provide an avenue for young athletes’ development in physical and psychosocial aspects. Athletes’ consistent development of competence, confidence, connection, and character (the 4 Cs) are the desired athlete outcomes for optimal development in a defined sport context, reflecting the extent of coaching effectiveness and the quality of youth sport programming. The purpose of this study was to assess athletes’ 4 Cs with respect to their age, gender, competitive level, and birthdate.

**Methods:**

Athletes (*n* = 454; 12–18 years of age) and their coaches (*n* = 45) from boxing, judo, karate, kickboxing, taekwondo, and wrestling completed validated measures of the 4Cs of athlete development. Analyses were conducted for each sport outcome separately to examine differences in athletes’ responses. A descriptive account of the athletes’ responses and univariate statistics (e.g., one-way ANOVAs) were used to assess participants’ responses.

**Results:**

Results revealed that the athletes scored high in each outcome. According to the comparisons, the older group of athletes had significantly higher competence and connection scores. Girls perceived less confidence than boys. Competence scores of athletes at local and international competitive levels differ significantly, with scores increasing with competitive level, while a significant decrease in perceived character was observed. Moreover, the athletes born in the first quartile of a year had significantly lower character scores.

**Discussion:**

Findings suggest that the athletes have generally gained positive attributes across the examined outcomes. However, significant differences observed in the athletes’ examined characteristics indicate areas for improvement in coaching practices and youth sport programming. The findings were discussed in light of the extant coaching literature, and suggestions for future studies were provided.

## Introduction

Sport practice is a crucial mechanism to create an ideal context for youth development in various settings (e.g., Camiré et al., 2009; Fraser-Thomas et al., 2005; Koh et al., 2017) including competitive sport (Strachan et al., 2011). Engaging in sport has the potential to provide youth with various developmental opportunities in physical, psychological, and personal aspects of development (Côté et al., 2024). Young athletes can develop physical health, specific motor skills, and athletic performance by participating in sports (Fraser-Thomas et al., 2005) while acquiring critical life skills (e.g., self-esteem, goal setting) that may be transferred (e.g., Koh et al., 2014). Researchers suggest that sport involvement should build strengths in youth by adopting a holistic view of athlete development to achieve sustainable success in sport (Côté and Gilbert, 2009). In sport psychology literature, these strengths, which has been adapted from developmental psychology (Lerner et al., 2005), have been conceptualized as young athletes’ development of competence, confidence, connection, and character through their consistent engagement in sport over time (e.g., a season-long participation) (the 4 Cs; Côté and Gilbert, 2009).

Côté and Gilbert (2009) defined that competence is an athlete’s sport-specific ability, comprising technical, tactical, and performance skills, along with improved health, fitness, and healthy training habits. Confidence refers to an athlete’s internal sense of overall self-worth (belief in one’s abilities). Connection refers to the quality of relationships that athletes have with coaches and people within and outside of their immediate sport setting. Character encompasses the athletes’ morality (respect for the sport and others), integrity, empathy, and responsibility, reflecting the demonstration of prosocial behaviors and avoiding antisocial ones. The presence of the 4 Cs in a sport context reflects itself as athletes perceiving themselves competent in their technical, tactical, and physical skills; confident in their capabilities; connected to their immediate surroundings, primarily their coaches and peers; and generating a stronger sense of character in future (Miller and Siegel, 2017). The 4 Cs model provides a framework for understanding the outcomes of sport participation while promoting its benefits. The extent to which athletes develop these outcomes is the primary indicator of coaching effectiveness and indirectly youth sport programming success (Côté and Gilbert, 2009; Vierimaa et al., 2012). Coaching effectiveness refers to the coach’s sustained use of integrated professional, interpersonal, and intrapersonal knowledge to foster athletes’ competence, confidence, connection, and character within their specific coaching environment (Côté and Gilbert, 2009). From this perspective, athletes’ perceptions of their 4Cs are direct reflections of coaches’ instructional behaviors, relational strategies, and the quality of the learning environment they create. This study aims to assess the developmental sport outcomes of young athletes in competitive contact sports and martial arts using the 4 Cs model.

Research in youth development in sport indicates that sport participation can improve athletes’ development across the lifespan (Armour et al., 2013; Fraser-Thomas and Harlow, 2024) while the idea that sport involvement generally facilitates sport development is flawed (Bruner et al., 2022). Recognized athlete development models (e.g., the Developmental Model of Sport Participation; the DMSP; Côté et al., 2016) offer sport stakeholders (e.g., coaches, coach development administrators) guidance for creating congruence between coaches’ knowledge and skills and athletes’ holistic developmental needs (i.e., the 4 Cs), based on their developmental age and competitive level. Drawing on an ecological view of human development (e.g., Bronfenbrenner, 1999), researchers suggest the Personal Assets Framework (PAF, Côté et al., 2014) to understand the benefits of sport involvement and the possible pathways for optimizing physical and social environments to foster youth athletes’ sport outcomes.

PAF suggests that (a) personal engagement in activities in which the specific activities young athletes participate, (b) the nature of relationships athletes form (quality relationships), and (c) the suitability of the physical and competitive sport environments (appropriate settings) form the quality of young athletes’ sport involvement. Among the specific factors directly influencing sport involvement from this perspective are athletes’ age, gender and type of sport they participate (e.g., Musch and Grondin, 2001; Agans and Geldhof, 2012; Culver and Kraft, 2020; Culver and Werthner, 2012), a balanced blend of practice and play activities as defined in the DMSP (Côté et al., 2014), coaches’ knowledge (Gilbert and Côté, 2013) including their repertoire of instructional strategies and teaching methods (e.g., Bean and Forneris, 2016; Kılıç and İnce, 2023), their accumulated athletic and coaching experience and formal education (e.g., Piggott, 2012), which involves undeliberate and deliberate learning (Trudel and Gilbert, 2024), the equity of their gender in the field (e.g., Culver et al., 2022; Townsend et al., 2024), and physical and psychological appropriateness of sport setting to athletes’ developmental needs (e.g., Bean et al., 2018; Strachan et al., 2011). Ideal dynamic interactions between these aspects nurture athletes’ 4 Cs, the building blocks that give rise to more enduring and overarching outcomes of sustainable sport participation, healthy performance development, and the development of critical life skills (Côté et al., 2024).

When reaching peak performance and scoring at early ages is on the forefront, though it is generally unnecessary (Mosher et al., 2020), sport programming and parallelly coaching practices may become shortsighted and tend to impose early selection and winning at the expense of other essential developmental aspects (i.e., personal development and sustainable participation; Côté et al., 2024) derailing from the path of holistic development (Camiré, 2015). Such practices in various sport contexts (e.g., school sport, Cobley et al., 2008) result in a considerable divergence from facilitating appropriate developmental opportunities for young athletes’ sport outcomes (i.e., the 4 Cs), a critical element for reaching excellence in coaching (Côté and Gilbert, 2009). Research in athlete development has long been documenting numerous negative physical (injuries, Elliot et al., 2010; health problems, Moseid et al., 2023) and psychosocial (Gencer, 2023; Strachan et al., 2009) athlete experiences and consequences of youth sport participation, most likely leading to athletic dropout during adolescence (e.g., Wall and Côté, 2007).

While several studies explored coaching effectiveness from athletes’ perspectives focusing on several aspects (e.g., Camiré et al., 2009; Boardley et al., 2008), other studies utilize the 4 Cs model to obtain a unified understanding of coaching effectiveness and the quality of sport involvement in youth sport. Allan and Côté (2016) explored the relationship between athletes’ 4 Cs and their coaches’ emotions in competitive youth soccer. The authors observed coaches to determine their emotional behaviors and collected data on the 4 Cs from their athletes. They found that calm and inquisitive coaching is related to athletes expressing more prosocial behaviors and less antisocial behaviors toward opponents than intense, hustling coaching, highlighting the association between coaches’ emotions and athletes’ moral development. Vierimaa et al. (2018) observed athletes’ behaviors during basketball competitions and compared them with their perceptions of the 4 Cs. The authors reported that the high Cs group communicated more frequently with their coaches, underlining the importance of coaches’ primary role in athletes’ developmental experiences.

Kassim and Boardley (2018) explored the extent to which coaches’ perceived effectiveness predicts athletes’ 4 Cs in a variety of team and individual sports participants from the UK and Malaysia. The authors found that athletes’ perceptions of their coach may be critical for their continued involvement in sport. The coaches’ perceived motivation effectiveness, technique effectiveness, and character-building effectiveness positively predicted athletes’ 4 Cs. To understand the extent of the presence of the 4Cs in competitive youth football, Santos et al. (2019) collected qualitative data (interviews and observations) from 19 adolescent athletes from Portugal. The authors found that while generally positive interactions among coaches and athletes have been observed, the psychosocial aspect of the 4 Cs model has been neglected. Adopting a longitudinal perspective, Erickson and Côté (2016) examined the relationship between athletes’ 4 Cs and the intervention tone of coaches’ behavior in youth volleyball teams by coding coaches’ interactions and matching with each athlete’s 4 Cs score in a continuum. Findings underscored the idiosyncrasy of coaches’ influence on young athletes, even when they are teammates and exposed to similar coaching.

In the current study context, approximately one-fourth of the registered athletes actively participated in sports during the last decade (Turkish Directory of Youth and Sports Statistics, 2024). Despite the growth in the number of sports clubs and registered athletes, research indicates that the active sport participation rate has been decreasing among adolescents (e.g., Kin-Isler et al., 2009), including high school sports (Pehlivan, 2013). Research on coaching practices, athletes’ outcomes, and the quality of sport settings points to several structural issues that may provide explanation to this downward trend. Regarding coaching practices, Yapar and Ince (2014) conducted an observational study in competitive youth basketball with the lens of the DMSP and found an incongruence between coaches’ practices and athletes’ context-specific developmental needs. Another line of research suggests that coaches’ early talent selection based on athletes’ chronological age may exclude potential athletes coaches deem untalented, narrowing developmental pool (e.g., Kılıç and Yılmaz, 2022; Söğüt et al., 2023). Furthermore, few recent studies specifically revealed areas of need in coaches’ pedagogical knowledge and capacity to provide a wide array of instructional strategies and teaching methods that resonate with athletes’ learning needs (Demiral and Nazıroğlu, 2024; Kılıç and İnce, 2023).

In relation to athlete outcomes, recently, Kilic and Ince (2021) examined young athletes’ 4 Cs in various individual and team sports adopting a program evaluation perspective. The authors reported a downward trend in the athletes’ 4 Cs scores as they age. Moreover, significant differences between genders were observed in competence, connection, and character outcomes. Girls scored lower in competence, whereas boys had lower scores in connection and character outcomes. One work explored the physical and psychological suitability of a youth gymnastics setting for holistic athlete development (Kılıç, 2023). The findings revealed areas for improvement for optimal athlete development. Collectively, these findings suggest that youth sport provision in Türkiye has been facing difficulties in providing optimal developmental context for sport development.

Within this broader landscape, exploring youth sport development in contact sports and martial arts are particularly noteworthy. The literature suggests, contact sports and martial arts can foster youth development by cultivating psychosocial attributes (Rodrigues et al., 2024) such as decreased anxiety, self-confidence, resilience (Massey and Whitley, 2024), lower aggression, self-reliance (e.g., Hackney, 2013), and compassion (Clapton and Hiskey, 2020), alongside physical excellence. However, in youth contact sport and martial arts, existing research often takes a unidimensional approach to assessing athlete outcomes, focusing mainly on technical, physiological, or anthropometric factors, predominantly within male- samples (e.g., Nakayama and Izawa, 2025). To date, little is known about how participation in contact sports and martial arts supports or hinders athletes’ holistic development. There has been a long-standing tradition of youth participation and sustained national and international competitive success in Turkish contact sports and martial arts, particularly wrestling, taekwondo, karate, judo, boxing, and kickboxing. Given the large number of young participants in contact sports and martial arts in the study context, a multidimensional assessment of their sport outcomes can provide critical insights into the strengths and areas for improvement in coaching practices and youth sport program design. Research investigating athletes’ holistic development in contact sport and martial arts is scarce. Accordingly, this study aimed to examine young athletes’ perceived competence, confidence, connection, and character across competitive contact sports and martial arts. Specifically, the study investigated whether the athletes’ perceived 4 Cs differ by their age groups, gender, competitive level, and date of birth.

## Method

### Participants

Athletes (*n* = 454) and their coaches (*n* = 45; 3 women, 42 men) from 45 sports teams in Ankara, Eskişehir, Malatya, Yozgat, and Kırşehir cities in Turkey participated in this study. Ankara is a large metropolitan city while Malatya and Eskişehir are middle-sized, and Kırşehir and Yozgat are small-sized, representing western, middle and eastern regions of Turkey. The inclusion criteria for participation were that the coaches had been training their team for at least 1 year and had obtained at least a second-level national coaching certificate, which equates to competition-development sport context or higher in Turkey. The age range of the coaches was between 24 and 62 (*M* = 39.4) and their average years of coaching experience was 14 (SD = 9.15). The athletes were between 12 and 18 years of age (*M* = 14.38, SD = 1.89), with an average of 4.48 years of sport experience (SD = 3.19). Detailed demographics of the athletes, based on age group, gender, competitive level, and birthdate, are presented in Table 1.

**Table 1:** Athletes’ demographics.

Characteristics	Athletes (n)	Percentage (%)
Age-group		
1 (12–14 years)	254	55.9
2 (15–18 years)	200	44.1
Gender		
Girls	233	51.3
Boys	221	48.7
Sports		
Boxing	34	7.5
Judo	58	12.8
Karate	25	5.5
Kickboxing	85	18.7
Taekwondo	180	39.6
Wrestling	72	15.9
Competitive level		
Local	111	24.4
Regional	81	17.8
National	189	41.6
International	73	16.1
Type of support program		
TOHM*	21	4.6
SEM*	38	8.4
No Support	395	87
Birthdate (Quartiles of a year)		
Q1	126	27.8
Q2	125	27.5
Q3	122	26.9
Q4	81	17.8

*TOHM: Olympic Athlete Development Scheme; SEM: Talent Selection and Development Scheme.

TOHM (Turkey Olympic Preparation Centers) and SEM (Athlete Training Centers) are two key programs in Turkey that aim to develop and train athletes. The main difference is that the SEM program focuses on younger athletes, aiming to discover talent and provide basic training, whereas the aim of the TOHM program is to select and train talented athletes with a high probability of participating in the Olympics, also systematically supporting their formal education and psychosocial development.

### Data collection instruments

The psychometrically tested forms of the scales suggested in the PYD Toolkit (Vierimaa et al., 2012) were applied to collect the data (Table 2). The PYD Toolkit enables capturing multiple constructs that evaluate youth athletes’ interrelated sport-specific outcomes across physical, mental, social, and emotional aspects. Specifically, the toolkit examines athletes’ perceived competence, confidence, connection, and character in the sport context. While helping pinpoint areas for development in coaching knowledge and practices, such program evaluation from the athletes’ perspective also enables the generation of proxy measure for the effectiveness of the respective sport programs the athletes have been participating in (Vierimaa et al., 2012). The multidimensional psychometric robustness of the PYD Toolkit has been recently proven (Chan et al., 2025). The findings of the cognitive interviews with athletes regarding the comprehensibility of measurement items suggest that the children aged 12 and above can be eligible for completing the measures (Kılıç and İnce, 2016).

**Table 2:** Measures and raters [Retrieved from Kilic and Ince (2021)].

Outcomes	Measurement	Source	Adaptation	Rater
Competence	Sport Competence Inventory	Adapted from Dunn et al. (2007)	Kılıç and İnce (2016, 2017a)	Self, coach, peer
Confidence	Self-Confidence Subscale of CSAI-2R	Adapted from Cox et al. (2003)	Kılıç and İnce (2016, 2017b)	Self
Connection	The Coach-athlete Relationship Questionnaire (CART-Q)	Jowett and Ntoumanis (2004)	Altıntaş et al. (2012) and Kılıç and İnce (2016)	Self
Character	The Prosocial and Antisocial Behavior in Sport Scale (PABSS)	Kavussanu and Boardley (2009)	Balcikanli (2013)	Self

The adapted version of the Sport Competence Inventory (Kılıç and İnce, 2016, 2017a), which provides a multi-perspective approach, was applied to measure the athletes’ competence. Accordingly, athletes, their coaches, and teammates completed the respective versions of the inventory for each athlete of the team examined. The competence score is rated on a 5-point Likert scale, ranging from 5 (extremely competent) to 1 (not competent at all), in terms of technical, tactical, and physical skills. The internal consistency value of the adapted version of the Sport Competence Inventory was 0.81 for athletes, 0.89 for coaches, and 0.92 for teammate ratings. For the present study, the internal consistency values were 0.77 for the athlete, 0.90 for the coach, and 0.93 for the teammate.

The athletes’ confidence was examined using the adapted version of the self-confidence subscale of the Revised Competitive State Anxiety-2 (Kılıç and İnce, 2016, 2017b). The subscale consists of five items (e.g., “I am confident because I mentally picture myself reaching my goal”) through which athletes rated their confidence on a 4-point scale, ranging from 4 (very much so) to 1 (not at all). The construct validity values of the scale were observed as good, with an internal consistency value of 0.76. For the present study’s data, the Cronbach’s alpha value for the subscale was 0.80.

The athletes’ connection was examined using the adapted version of the Coach-Athlete Relationship Questionnaire (the CART-Q; Altıntaş et al., 2012). The CART-Q consists of 11 items (e.g., When I am coached by my coach, I am ready to do my best) to measure perceived coach-athlete relationship on a 7-point scale (7 = Extremely to 1 = Not at all). Altıntaş et al. (2012) reported Cronbach’s alpha values for the subscales of the CART-Q, ranging from 0.82 to 0.90, for athletes. For the present sample, the internal consistency values of the subscales were close to 0.80.

Character was examined using the adapted version of the Prosocial and Antisocial Behavior in Sport Scale (PABSS, Balcikanli, 2013). The PABSS consists of 20 items with four subscales that measure athletes’ prosocial (e.g., While playing for my team this season, I gave constructive feedback to a teammate) and antisocial behaviors (e.g., While playing for my team this season, I deliberately fouled opponent) that they demonstrate during training and competitions. The Cronbach’s alpha values of the subscales of the 20-item PABSS were reported to range from 0.70 to 0.72 (Balcikanli, 2013). For the present sample, the internal consistency values of the prosocial and antisocial subscales ranged from 0.67 to 0.78.

### Data collection procedures

After obtaining approval from the Kırşehir Ahi Evran University Social and Humanities Sciences Scientific Research and Publication Ethics Committee, the data, including specific demographic information (e.g., birthdate of an athlete), were collected by the lead author, who visited the club settings located in the respective cities. As competence measurement involves triangulated evaluation, the lead researcher prepared the PYD Toolkit specific to each team before site visits. Coaches and athletes completed the adapted versions of the PYD Toolkit measures. In each setting, data were collected separately from coaches and their athletes to ensure the trustworthiness of the responses. Before data collection, the athletes were instructed to fill out the PYD Toolkit considering their current specific context (i.e., sport club). The athletes completed the toolkit in approximately 20 minutes.

### Data analysis

Significant differences in the athletes’ outcomes in each sport, regarding their age group, gender, competitive level, and date of birth, were examined to answer the research questions. First, the descriptive information of the athletes’ responses for each outcome was reported. Then, the athletes’ outcome scores were compared using univariate, parametric, and non-parametric tests using SPSS 29. In examining age-based outcome score differences, the athletes were grouped into two age groups [Age-group 1 (12–14) and Age-group 2 (15–18) years of age], considering the age stage cutoff approximations in the DMSP (specialization and investment years; Côté et al., 2014). In analyzing the possible significant differences between the athletes’ chronological age and their perceived outcomes, their birth months were categorized into four quartiles of a year (i.e., 3-month periods), a method that has been widely used to examine relative age effects (e.g., Cobley et al., 2009).

### Data screening

Before conducting data analysis, the dataset was screened regarding missing data, and the applicability of one-way ANOVA (multivariate normality and homogeneity of variance). Besides, to ensure the normality assumption was met, a wide range of values in the data was checked (Pallant, 2007). Shapiro–Wilk values, histograms, and Q-Q plots were examined for each outcome score (Pallant, 2007). Mean values and 5% trimmed mean values were also observed close to the 4Cs scores, and the skewness and kurtosis values for each outcome score were between −2 and 2, except for connection scores. When it was considered all statistical values, histograms and Q-Q plots, it was observed that three dependent variables (competence, character, and confidence) were normally distributed, but one variable (connection) was violating the normality. For homogeneity of variance, the Levene test for competence, confidence, and character scores indicated equal variances (*p* > 0.05). Therefore, competence, confidence and character scores were analyzed using independent sample t-test and one-way ANOVA, whereas connection scores were analyzed using Mann–Whitney Test and Kruskal-Wallis’s test.

## Results

Examination of the descriptive account of the athletes’ responses revealed a general high outcome score in each of the 4 Cs. (Table 3). All of the athletes’ perceived outcomes fell within the upper quartile of their respective ranges.

**Table 3:** Descriptive account of the athletes’ sport outcomes.

Measures	Mean	SD	Min	Max
Competence (1–5)	3.68	0.64	1.83	4.93
Confidence (1–4)	3.30	0.56	1.00	4.00
Connection (1–7)	6.56	0.63	2.82	7.00
Character	4.16	0.40	2.6	5.0

Character score calculated by extracting antisocial score from the prosocial score.

The athletes’ 4 Cs scores, categorized by age groups (12–14 and 15–18) and gender, would be analyzed using independent sample t-tests. On the other hand, 4 Cs scores in terms of competitive level, and birthdate quartiles would be analyzed with one-way ANOVA. Furthermore, *post hoc* analyses were performed on all statistically significant ANOVA tests, and Bonferroni test results were interpreted. The results of the comparisons are reported separately below.

**Figure 1:**
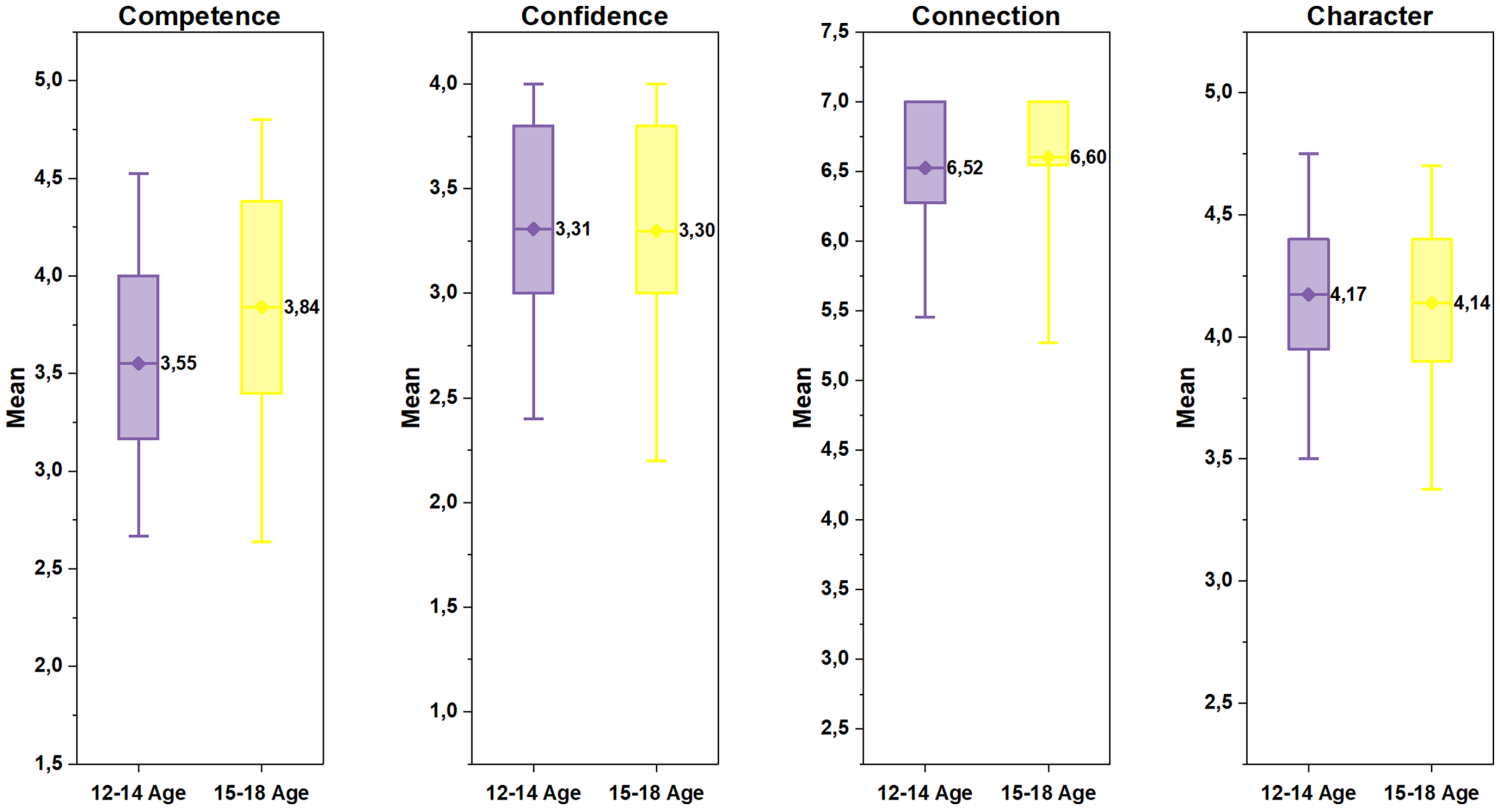
The athletes’ mean scores in each Cs by age-group.

Age group comparisons of the athletes’ 4 Cs revealed that there were no significant differences in the athletes’ character and confidence scores between age-group 2 and age-group 1 (character: *t*(452) = 0.91, *p* = 0.37, Cohen’s d = 0,086; confidence: *t*(452) = 0.18, *p* = 0.86, Cohen’s d = 0.017). However, scores of the age-group 2 in competence and connection were found significantly higher than those of their younger counterparts (competence: *t*(406) = −5.21, *p* < 0.001, Cohen’s d = −0.499; connection: *U* = 21257.5, *z* = −3.05, *p* = 0.002, *r* = −0.14). Figure 1 presents athletes’ 4 Cs scores based on age groups.

**Figure 2:**
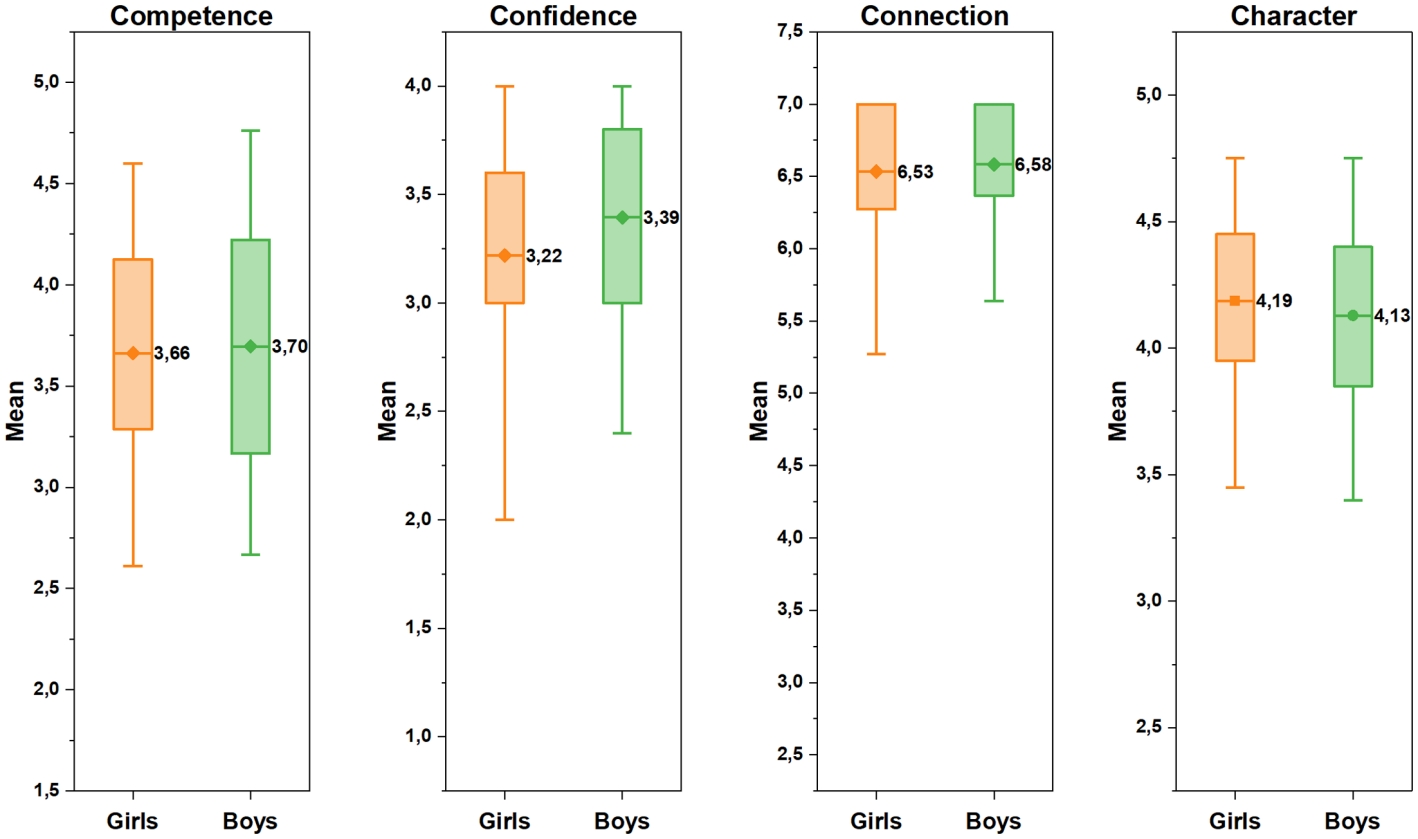
The athletes’ mean scores in each Cs by gender.

Gender-based comparisons of the athletes’ outcomes indicated that boys had significantly higher confidence scores than girls (*t*(452) = −3.38, *p* < 0.001, Cohen’s d = −0.32). No significant differences were found between boys and girls in terms of character scores (*t*(452) = 1.56, *p* = 0.119, Cohen’s d = 0.147), competence scores (*t*(452) = −0.54, *p* = 0.59, Cohen’s d = −0.05), and connection scores (*U* = 25,328, *z* = −0.31, *p* = 0.76, *r* = −0.014) as can be seen in Figure 2.

The findings of the comparisons on athletes’ competitive levels revealed significant differences between the athletes’ competence scores of local, regional, national, and international level athletes, each of which was found to be higher than the previous competitive level when moving backward from the international level by performing a post hoc Bonferroni Test (*F*(3, 453) = 56.72, *p* < 0.001, η^2^ = 0.27).

**Figure 3:**
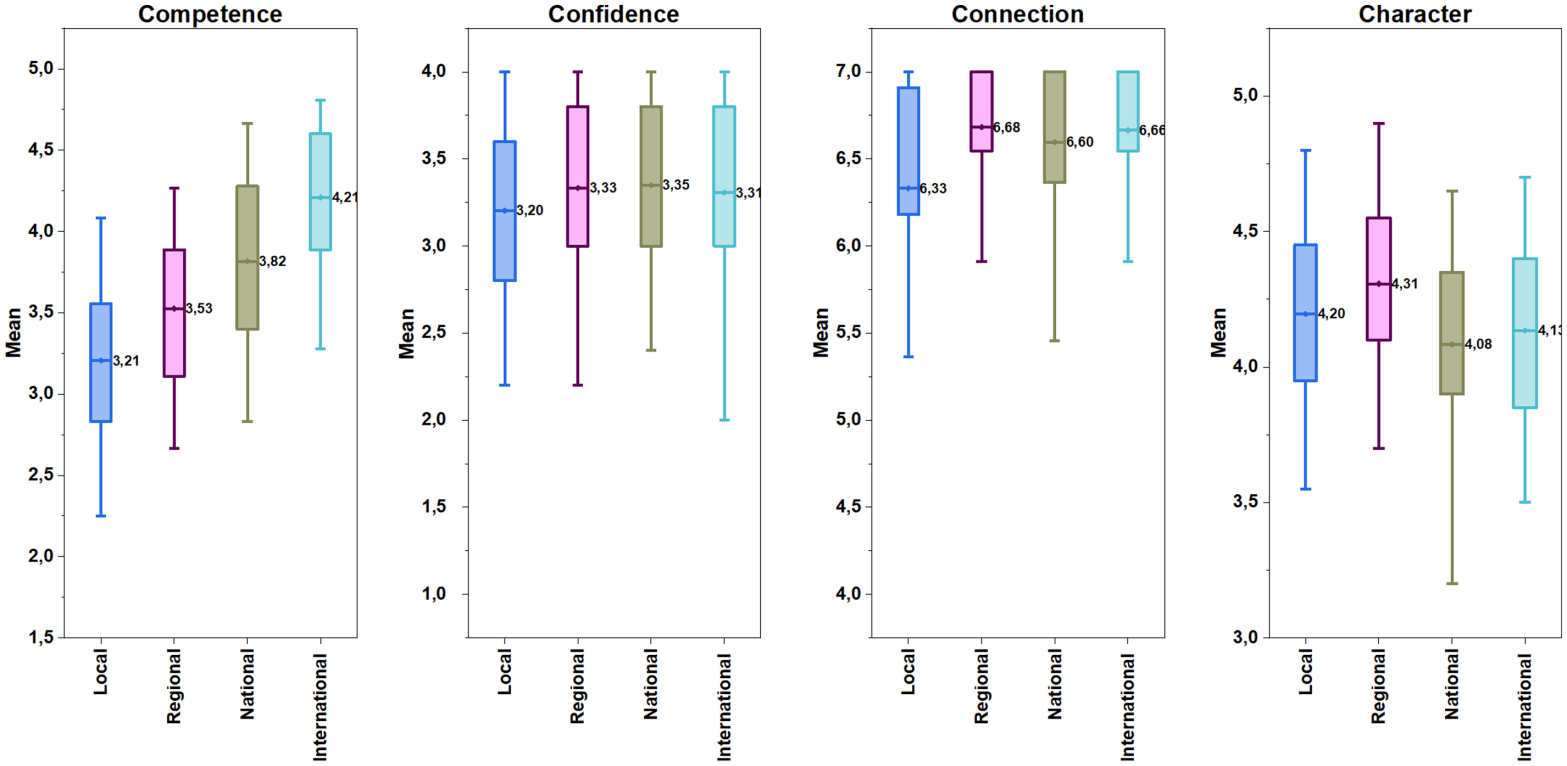
The athletes’ mean scores in each Cs by competitive level.

**Figure 4:**
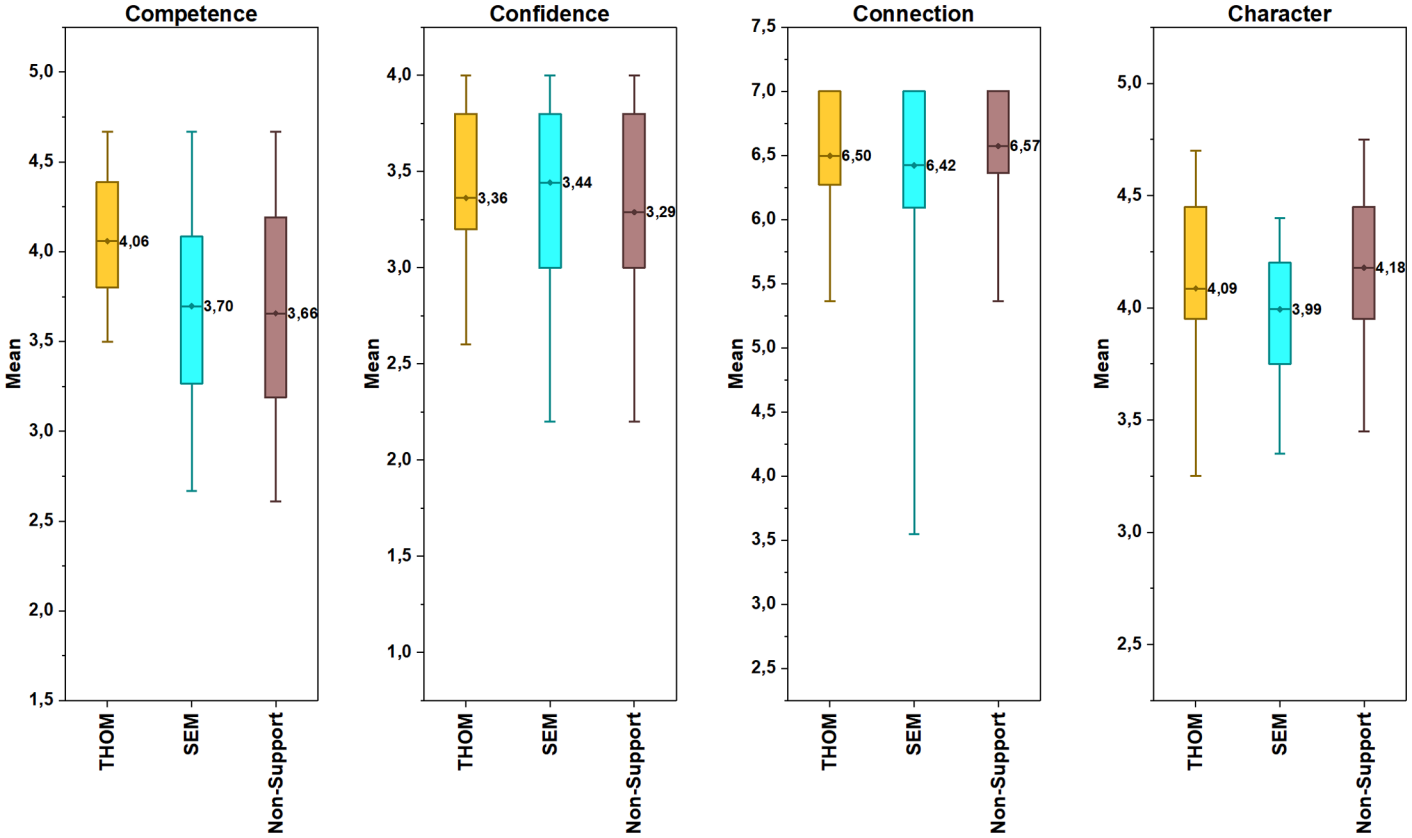
The athletes’ mean scores in each outcome by type of support program.

There was no significant difference in confidence scores of the athletes from different competitive levels (*F*(3, 450) = 1.71, *p* = 0.17, *η^2^* = 0.011). However, when character scores were examined, the scores of the regional-level athletes were significantly higher than those of their national and international-level counterparts (*F*(3, 450) = 6.75, *p* < 0.001, *η^2^* = 0.043). According to competitive level comparisons of athletes’ connection scores, it was observed that local-level athletes had the statistically lowest score among all levels (*χ*^2^ (3, 454) = 20.43, *p* < 0.001, *η^2^* = 0.04) (see in Figure 3). Findings about TOHM and SEM programs indicated that the athletes’ scores in the TOHM program were significantly higher than participants in the SEM program and no program in terms of competence scores (Welch *F*(2, 40.65) = 14.93, *p* < 0.001, *η^2^* = 0.025). On the contrary, non-support participants had scored significantly higher than SEM program participants in terms of character (*F*(2, 451) = 4.23, *p* = 0.015, *η^2^* = 0.018). However, the athletes’ scores in confidence and connection were not statistically different among programs (confidence: *F*(2, 451) = 1.43, *p* = 0.24, *η^2^* = 0.006; connection: *χ*^2^ (2, 454) = 0.881, *p* = 0.644). In Figure 4, the athletes’ mean scores in each outcome by type of support program is illustrated.

**Figure 5:**
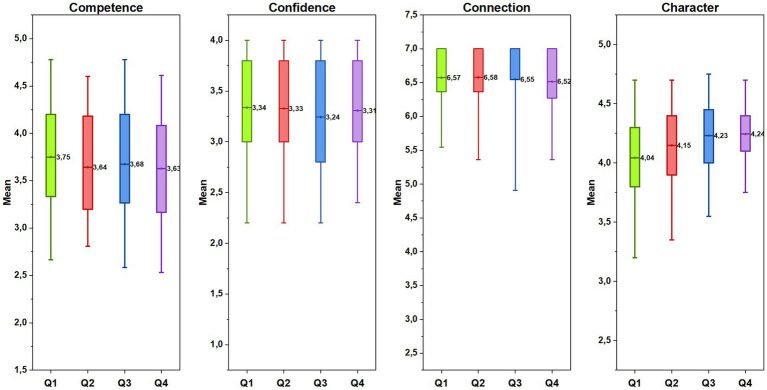
The athletes’ mean scores in each outcome by birthdate.

The findings on the comparison of the athletes’ outcomes by birthdate quartiles indicated that athletes born in Q1 scored significantly lower than athletes born in Q3 and Q4 in terms of character scores (Welch *F*(3, 243.48) = 6.69, *p* < 0.001, *η^2^* = 0.04). However, scores in competence, connection and confidence were not statistically different among quartiles (competence: *F*(3, 450) = 0.63, *p* = 0.59, *η^2^* = 0.004; connection: *χ*^2^(3, 454) = 2.34, *p* = 0.51; confidence: *F*(3, 450) = 0.68, *p* = 0.56, *η^2^* = 0.005). The athletes’ mean scores in each outcome by birthdate is illustrated in Figure 5.

## Discussion

One practical way to evaluating coaching effectiveness is to investigate athletes’ desired developmental sport outcomes from their own perspectives (Erickson et al., 2024). Drawing on the 4 Cs model (Côté and Gilbert, 2009), the current study examined the four domains of development in competitive youth contact sports among the athletes. Specifically, the athletes’ perceptions of competence, confidence, connection, and character, and whether their scores differ in each of them by their age, gender, competitive level, and birthdate were examined. In the following paragraphs, we discuss the implications of the findings with the extant literature, which we believe is relevant to coaching practice, coach development policies, and future research for young athletes’ holistic development in sport.

Researchers suggest that positive development through sport participation will manifest itself in the development of the 4 Cs (Côté and Gilbert, 2009; Vierimaa et al., 2012). Descriptive accounts of the athletes’ responses to each outcome may indicate that they have been gaining positive attributes through sport participation. The increase observed in the mean scores of the older group of athletes’ 4 Cs may indicate an enduring positive sport experience in the examined sport settings. From the PAF perspective (Côté et al., 2024), it may be argued to an extent that the athletes’ personal engagement in their sport activities (i.e., play and practice activities) has been nurturing, they build fostering relationships with others within the sport context, and the sport settings have been suitable for their physical and psychosocial development. The findings may support the view that contact sports and martial arts can indeed foster holistic developmental attributes (e.g., Hackney, 2013; Massey and Whitley, 2024; Rodrigues et al., 2024).

Comparisons of the athletes’ 4 Cs scores on age groups indicated a significant increase in the older group of athletes’ perceived competence and connection. The older (15–18 years) group of athletes coincides within the “specializing years” in the DMSP, where they begin to focus on practicing one sport with an increasing level of high-intensity year-round training (Côté et al., 2014). The athletes’ high level of perceived competence with an increasing trend may indicate their exposure to developmentally appropriate performance practices, including aligned instruction according to athletes’ learning needs (pedagogical knowledge; Trudel et al., 2013). Indeed, coaches need to be knowledgeable and have the capacity to provide technical corrections and feedback to develop athlete competence (Smith and Smoll, 2002). According to self-determination theory (Ryan and Deci, 2002), competence is nurtured by autonomy-supportive practices whereby the coaches’ use of praise and feedback, giving athletes choice, creation of a meaningful learning environment and positive relationships between teammates, and quality relationships with athletes are at the forefront (Vella et al., 2018). Athlete autonomy can be nurtured through appropriate instructional strategies and teaching methods that encourage athletes to take control of their learning and development processes (Kılıç and İnce, 2023). Coaching practices, including coaching pedagogy applied in the current study context, warrant further investigation for a more complete understanding of athletes’ competence development.

The 4 Cs scores of the older group of athletes were generally high; however, the younger group of athletes scored significantly less in connection. This may partially indicate the comparably low level of bonds between the athletes and their significant others (i.e., coaches and peers), characterized by fewer bidirectional exchanges between them. The older age group of athletes may have spent a longer time in a competitive context, during which they form close friendships and relationships that may, in turn, lead to stronger connections among athletes (Côté et al., 2010). Researchers also highlight the significance of coach behaviors in fostering athlete connection. There is a close relationship between a high-quality coach-athlete relationship and positive developmental experiences (Jowett, 2017; Smith and Smoll, 2007; Vella et al., 2011). Quality friendships are also critical to the development of connections for young athletes (Côté et al., 2010). Importantly, recent research suggests that athletes’ connection with their peers also has a positive relationship with their perceived competence (Erickson and Côté, 2016). Coaches’ active promotion of positive relationships with their athletes and friendship among teammates is paramount to developing healthy connections with athletes (Smith and Smoll, 2007; Côté et al., 2010).

Findings on gender comparison revealed that girls perceived themselves as significantly less confident than boys when performing their sport. This may be partially attributed to being disadvantaged in participating in sports, mainly due to the prevalence of gender discrimination in youth team sports (e.g., Var and Var, 2025) and contact sports and martial arts (e.g., Yaprak and Amman, 2009) in the study context. The rate of sport participation among girls and women was approximately 29% as of 2017 (Koca, 2018). Girls’ sport development is influenced by various factors, including their sport culture and national culture. Societal practices and cultural beliefs affect gender expectations in sport participation (LaVoi et al., 2007). Culver and Kraft (2020) suggest that the perpetuation of inequality and gendering language, driven by the androcentric model of sport, has a negative impact on girls’ development in sport. Coaches often contribute to this situation by their expectations and belief systems, resulting in negative perceptions of girls’ self-esteem during adolescence (Thapar et al., 2012). Culver and Werthner (2012) suggest that boys aim to establish their superiority in group play by showcasing their skills and knowledge. In contrast, girls focus on social interaction and reducing their rank compared to others. Therefore, girls may require considerable reassurance and support in developing sport confidence, particularly at a young age when they may have limited exposure to success. Further exploration of the underlying ecological reasons for girls’ lower perceived confidence is needed, with adopting social justice perspective (Camiré et al., 2022).

Comparisons based on the athletes’ competitive level indicate that their perceived competence significantly increases as they progress from local to national and international levels of competition. This trend has also been observed in the degree of receiving professional support (e.g., being a TOHM athlete). The athletes’ perceived competence significantly increased from being non-supported to being belonged to a SEM and a TOHM schemes, respectively. It appears that athlete satisfaction with competence increases as they progress from local to international levels and when they become a member of a national athlete development scheme. Perception of competence is critical for young athletes to determine their own place in sport participation (Agans and Lerner, 2021). Social settings that support the satisfaction of competence are more likely to nurture growth and intrinsic motivation (Deci and Ryan, 2000). Higher perceptions of competence are linked to numerous outcomes, including high levels of achievement, increased self-esteem, and lower levels of anxiety (Côté et al., 2010). This finding might also indicate the effectiveness of the national athlete development schemes (i.e., TOHM) to an extent that they provide an optimal context for athletes’ development in sport.

While athletes’ outcome scores were generally higher among different competitive levels, the findings revealed that regional-level athletes scored significantly higher in character compared to their higher-level counterparts. This raises the question of whether a deterioration in the athletes’ character development might occur over time. Character development is an indispensable part of athlete development (Côté and Gilbert, 2009). Youth’s participation in competitive sport may enhance character development (Fraser-Thomas et al., 2005; Strachan et al., 2009), but coaches are one of the most influential factors in athletes’ life skill development including character (Gould and Carson, 2008). Coaches’ practices in fostering moral behavior, especially empathy and sportspersonship (e.g., Fraser-Thomas et al., 2005; Strachan et al., 2009), as well as prosocial behaviors and their antecedents (Kavussanu, 2025) in their athletes, warrant further consideration to better understand the athletes’ character development in the present study context.

Comparison of athletes’ outcomes with their birthdate quartiles revealed that athletes born in the first quartile had significantly lower perceived character than those born in the third and fourth quartiles, respectively. Sport participation shaped by chronological age can create relative age effects, which adversely affect athletes’ development in sport in the long run by creating a selection and training bias (e.g., Cobley et al., 2009). Research suggests that athletes born in the first quartile are generally labeled as “talented” based on their physical growth and development, and consequently receive extensive coaching support, intensive training, and competition opportunities (e.g., Hancock, 2020; Kons et al., 2025). This abundance in sport experiences may take an overwhelming form, resulting in physical exertion and exposure to greater levels of pressurized training and competitive conditions, such as being forced to play with their older peers (e.g., Hancock, 2020; Kılıç and Yılmaz, 2022). This may lead to an overemphasis on physical performance, which can create an ego-oriented mindset among athletes, resulting in low levels of moral functioning (Kavussanu and Ntoumanis, 2003). Playful sport environments underlined in the DMSP, instead, can foster character development in competitive sports, especially empathy and caring (Côté et al., 2010). Future studies should continue to trace the relationship between athletes’ 4 Cs and their birthdate distribution to further explore relative age effects from a developmental perspective (Kelly et al., 2021).

### Practical implications

The findings of the study highlight several areas for improving youth participation in competitive contact sports and martial arts. Accordingly, coaches should enhance athletes’ competence and connection, particularly among younger athletes, by implementing age-appropriate practices to increase interaction and intentionally structuring relational activities (Côté and Gilbert, 2009). Coaches’ inclusive, autonomy-supportive (e.g., Pill et al., 2022), and gender-sensitive practice to girls’ sport participation (Culver and Werthner, 2012) will help effectively improve their sport confidence. Regarding character, coaches and clubs can integrate structured moral and prosocial learning opportunities into training to ensure healthy character development in sport. Their consistent 4 Cs evaluation of athletes to monitor coaching effectiveness (Vierimaa et al., 2012) can help inform policies to improve athletes’ holistic development in sport, reducing negative influences such as gender inequalities and relative-age effects. Athlete development schemes (i.e., TOHM and SEM) may also benefit from integrating holistic monitoring of athlete development into program design and coach development, expanding coaching requirements for a sexually, developmentally, and contextually appropriate athlete development approach within their systems, and encouraging continuous learning for coaches (Trudel and Gilbert, 2024).

### Limitations

Despite these promising findings, it is acknowledged that the current study, through the method employed, presents the athletes’ perceived outcomes within specific settings of Turkish youth sport at a particular point in time. Thus, the confirmation of the athletes’ positive responses was not possible, and the interpretation of the findings is constraint to Turkish context. This said, athletes’ (and partially their coaches’) self-reported 4 Cs enabled the generation of data for investigating young athletes’ holistic development in contact sport and martial arts (Vierimaa et al., 2012). The 4 Cs model provided a tool for comparing between groups of athletes (Erickson et al., 2024). Youth development in sport is generally researched in terms of outcomes and experiences in actual or perceived conditions (MacDonald et al., 2024). The 4 Cs model provided clear boundaries (Erickson et al., 2024) with a robust statistical structure (Chan et al., 2025) for understanding the athletes’ desired outcomes in physical and psychosocial aspects of sport. It also enabled the drawing of clear conclusions from the research findings, while avoiding the examination of a multitude of variables and the challenge of measuring a range of constructs. Another potential limitation concerns the risk of self-selection bias – where individuals who are more inclined to participate disproportionately contribute to the data – which may lead to overly optimistic self-reporting (MacDonald et al., 2024). Additionally, social desirability may further amplify self-report bias, resulting in positively skewed responses (van de Mortel, 2008). To eliminate these limitations, the lead researcher visited each sports setting, include a broad and diverse sample of athletes in each location, and administered the measures independently from their coaches. Importantly, the athletes’ self-report of a decreased 4 Cs with age in a previous study in the same sport culture (Kilic and Ince, 2021) brings up a question of the extent of applicability of self-selection bias for different sport contexts. However, future studies should continue to explore youth sport outcomes and design longitudinal studies that apply methodological triangulation (e.g., observation data) to generate more robust understanding of athletes’ outcomes in various sport settings.

## Conclusion

This study exemplifies a multidimensional assessment of young athletes’ developmental sport outcomes in competitive contact sports and martial arts using the 4 Cs model. The psychometrically tested version of scales suggested in the PYD Toolkit enabled investigating athletes’ sport competence, confidence, connection, and character in relation to their critical demographic characteristics. Recent research has utilized the 4 Cs model in various ways to investigate young athletes’ 4 Cs. In this study, the 4 Cs model was employed to assess coaching effectiveness in competitive youth contact sports and martial arts. Future studies should continue to expand on the various applications of the 4 Cs. Such studies should consider incorporating athlete experiences (MacDonald et al., 2012) in addition to their 4 Cs within a longitudinal study design to more effectively assess how coaching practices (and adjacent contexts) impact young athletes’ sport involvement upon which effective intervention programs can be developed (Erickson et al., 2024; MacDonald et al., 2024). To our knowledge, one study assessed the impact of an intervention program, designed to develop coaches’ knowledge and practices in facilitating athletes’ 4 Cs in competitive youth sports (Kılıç et al., 2024). As such, the 4 Cs model deserves further use along with examining athletes’ sport experiences to generate stronger evidence for the quality of coaches’ practices and youth sport programming. Such knowledge can inform future intervention studies that encourage coaches’ collaborative learning whereby their curiosity and lifelong learning flourish (Culver et al., 2024).

## Data Availability

The raw data supporting the conclusions of this article will be made available by the authors, without undue reservation.
