# Correction: Coaching effectiveness in competitive youth contact sports and martial arts: athletes' and coaches' perceptions

**DOI:** 10.3389/fpsyg.2026.1788820

**Published:** 2026-02-05

**Authors:** İlayda Müjdeci, Koray Kılıç, Aykut Bulut, Hakan Kuru

**Affiliations:** 1 Faculty of Sport Sciences, Ahi Evran University, Kırşehir, Türkiye; 2 Faculty of Education, Ahi Evran University, Kırşehir, Türkiye; 3 Faculty of Sports Sciences, Istanbul Rumeli University, İstanbul, Türkiye

**Keywords:** athlete development, program evaluation, relative age effects, the 4 Cs, youth sport

Author Hakan Kuru was erroneously assigned to affiliation Faculty of Sports Sciences, Technische Universiteit Delft, Delft, Netherlands. This affiliation has now been removed for author Hakan Kuru.

Affiliation Faculty of Sports Sciences, Istanbul Rumeli University, İstanbul, Türkiye was omitted from the paper. This affiliation has now been added for author Hakan Kuru.

The original version of this article has been updated.

